# P-667. The Impact of Delay in the Start of Effective Antibiotics on the Course of *Pseudomonas aeruginosa* Pneumonia: a Retrospective Study

**DOI:** 10.1093/ofid/ofae631.864

**Published:** 2025-01-29

**Authors:** Takanori Ito, Osamu Kanai, Kohei Fujita, Kiminobu Tanizawa

**Affiliations:** Kyoto Medical Center, Kyoto, Kyoto, Japan; National Hospital Organization Kyoto Medical Center, Kyoto, Kyoto, Japan; National Hospital Organization Kyoto Medical Center, Kyoto, Kyoto, Japan; Kyoto Medical Center, Kyoto, Kyoto, Japan

## Abstract

**Background:**

The decision to target up to *Pseudomonas aeruginosa* at the start of antibiotic treatment of bacterial pneumonia is a major turning point. Since *P. aeruginosa* itself is a mildly virulent bacterium, we experience that *P. aeruginosa* pneumonia is improved even targeting *P. aeruginosa* after its detection. We studied the differences in mortality among *P. aeruginosa*pneumonia cases depending on whether antibiotics with anti-*P. aeruginosa* activity were administered from the first time (empirical group, group E) or after the detection of *P. aeruginosa* (definitive group, group D).

**Methods:**

We retrospectively studied patients who received antibiotics with anti-*P. aeruginosa* activity for *P. aeruginosa* pneumonia at our hospital from October 2016 to December 2023. The primary endpoint was 30-day mortality, and the secondary endpoints were total duration of treatment, length of hospital stay, and adverse events. This study was conducted with the approval of the Ethics Committee of our hospital (approve number: 23-075).

**Results:**

There were 95 applicable participants. The group E had 62 patients and the group D had 33 patients. In the background between the two groups, clinical frailty scale was significantly worse, and impaired consciousness and hypotension. The 30-day mortality was not significantly different between the two groups (group E vs. group D, 1.6% vs. 3.0%, p > 0.99). And there were also no significant differences in mean total duration of antibiotics treatment, mean length of hospital stays, or incidence of adverse events between the two groups (13.2 days vs. 12.9 days, 83.8 days vs. 58.5 days, 54.8% vs. 63.6% in the group E vs. group D, respectively).

**Conclusion:**

There was no statistical difference in mortality by the delay in targeting *P. aeruginosa* for treatment of *P. aeruginosa*pneumonia. It was suggested that this could lead to appropriate use of broad-spectrum antibiotics that target even *P. aeruginosa*.

**Disclosures:**

**All Authors**: No reported disclosures

